# Quantification of soil microbial functional genes as potential new method in environmental risk assessment of pesticides

**DOI:** 10.1007/s10646-025-02920-w

**Published:** 2025-06-27

**Authors:** Fabian Stache, Franziska Ditterich, Zuzana Hochmanová, Jakub Hofman, Christian Poll, Ellen Kandeler

**Affiliations:** 1 Institute of Soil Science and Land Evaluation, Soil Biology Department, University of Hohenheim, Stuttgart, Germany; 2 RECETOX, Faculty of Science, Masaryk University, Brno, Czech Republic

**Keywords:** OECD 216, phoN, Boscalid, Non-target soil microorganisms, Pesticide risk assessment

## Abstract

Pesticides can have adverse effects on soil microorganisms, but they are underrepresented in the currently required OECD 216 test for environmental risk assessment of plant protection products (PPP). The guideline monitors soil microbial nitrogen transformation over 28 days, potentially missing long-term effects of persistent pesticides. Additionally, nitrate alone may be not sensitive enough to detect disruptions in microbial functions. We investigated whether functional gene analysis could provide a more sensitive bioindicator of pesticide impact. To compare this method with the standard test, we conducted a microcosm experiment following the OECD 216 experimental setup. To capture long-term effects beyond the typical test period, we extended the incubation duration to 56 days. Four different concentrations of the persistent fungicide boscalid were added based on predicted environmental concentration. We also assessed microbial responses to fungicide exposure by measuring classical soil microbial parameters. According to the standard test, boscalid had no harmful long-term effects on soil microbiota. In contrast, our analysis of functional genes found an overall reduction in the acid phosphatase-encoding *phoN* gene abundance on Day 56, and correspondingly, in acid phosphatase activity in the highest fungicide treatment. Simultaneously, we observed a tendency towards lower fungal abundance based on measured copy numbers of an ITS region of nuclear ribosomal DNA (rDNA) and increased cumulative CO_2_ production. These results indicate a fungicide-related response of the microbial community and impaired microbial phosphorus cycling. Extending the experimental period to 56 days revealed long-term effects that would have otherwise been undetected under the typical 28-day test duration.

## Introduction

Soils are complex and dynamic natural systems serving as habitats for a vast diversity of organisms, crucial for fulfilling various ecosystem functions such as nutrient cycling, plant productivity, and carbon (C) storage (Smith et al. 2015; Pereira et al. 2018). However, the extensive use of agrochemicals in modern agriculture threatens to disrupt these microbial communities and their functions (Meena et al. 2020; Steiner et al. 2024). While plant protection products (PPP) are intentionally used in agricultural ecosystems to ensure plant yield and quality, they can harm non-target organisms. Fungicides, for instance, can alter microbial community structure (Ma et al. 2021), decrease soil enzyme activities (Deborah et al. 2013; Baćmaga et al. 2016), and impact the abundance of functional genes involved in nitrogen (N)-cycling (Han et al. 2022). Despite the well-known role of soil microorganisms in soil functioning, their ecotoxicological responses are not sufficiently represented in the PPP regulatory framework and are limited only to the N transformation test (OECD 216).

The introduction of new PPP and new active substances to the European market is governed by regulatory framework based on Regulation 1107/2009 (EU 2009), which specifies that active substances are only approved and PPP are only authorized if their ecotoxicological effects can be minimized or completely avoided. To achieve this, active substances and PPP undergo an environmental risk assessment (ERA) guided by the European Food Safety Authority (EFSA). In this process, standardized ecotoxicity tests are used to validate the safety for organisms across different trophic levels and habitats. While different ecotoxicological tests exist for soil fauna and terrestrial plants, soil microorganisms remain underrepresented in the current testing framework. This is concerning, given that the EFSA emphasized the specific goal of protecting soil microbes to prevent adverse effects on ecosystem functioning (EFSA 2010). Despite this, the ERA for soil microorganisms continues to rely only on Test No. 216 Soil Microorganisms: Nitrogen Transformation Test of the Organization for Economic Co-operation and Development (OECD 2000). This test was designed to detect long-term effects of chemical or natural substances on N transformation, by measuring nitrate concentrations in treated and untreated soils for a minimum of 28 days. If no deviation greater than 25% between untreated and treated soil is observed by Day 28, the test can be stopped. Otherwise, the test continues until the deviation shrinks below 25% or the duration reaches a maximum of 100 days. The guideline has received criticism for potentially overlooking the harmful effects of pesticides on soil microbiota (Thiour-Mauprivez et al. 2019; Pedrinho et al. 2024). Additionally, the test’s duration may not fully capture the long-term effects of pesticides, whose half-lives (DT_50_) can vary widely, from a few days to months or even years (Wang et al. 2018; Šudoma et al. 2019). Moreover, in the current approach, it is considered correct when the test is not prolonged if effects don’t exceed 25%, while the effects that might evolve after 28 days are neglected.

There have been ongoing discussions about new methodological approaches for testing soil microorganisms, especially considering the advancements in laboratory methods over the last decades. In its most recent scientific statement regarding the ERA for soil organisms, EFSA (2017) addressed the shortage of standardized tests for soil microorganisms and recommended the inclusion of arbuscular mycorrhizal fungi (AMF) as a new biomarker. Sweeney et al. (2022) reviewed the viability of AMF as a new biomarker and suggested that the spore germination test may be a reliable option for a sensible first-tier test. However, they disagreed with EFSA’s suggestion to include AMF community metrics in risk assessment, as knowledge gaps remain on the link between functional diversity and phylogenetics of AMF. Another approach was proposed by Romdhane et al. (2019), who used the specific enzyme targeted by the herbicide β-triketone as a possible new biomarker. In addition, newer molecular methods, such as amplicon sequencing, were suggested by Karpouzas et al. (2022). While amplicon sequencing can characterize the composition of bacterial, fungal and archaeal soil communities, it does not provide direct information about their functional roles (Schöler et al. 2017). To bridge that gap, analysis of functional genes could offer insights into the genetic potential of soil microorganisms to perform specific functions. Functional genes have already been used as proxies to estimate nitrous gas emissions from soils (Morales et al. 2010) or to investigate the effects of different pesticides on soil C and N cycling (Fang et al. 2020; Sim et al. 2022). The genes for ammonia oxidizing bacteria and archaea (*amoA* AOB/AOA) were also recommended as new potential biomarkers for pesticide risk assessment (Karas et al. 2018). An advantage of using functional gene analysis is that the prerequisites for these analyses (DNA extraction and general procedures of qPCR) are already available as standardized ISO methods.

We aimed to evaluate the quantification of functional genes encoding N- and phosphorus (P)-cycling enzymes as a test procedure to assess the effects of pesticides on nutrient cycling in the soil microbial community and to compare its results with the existing OECD 216 test. Therefore, our experiment followed the OECD 216 guideline to allow a direct comparison between the molecular method and the standard test. Since the OECD test focused on N transformation, we selected key functional genes involved in N-cycling processes, including N-fixation (*nifH*), nitrification (*amoA* AOA/AOB) and denitrification (*nirK*/*nirS*) along with the *phoN* gene, which encodes an unspecific acid phosphatase in soil P-cycling. We used boscalid as it is one of the most widely used fungicides in Europe (Sabzevari and Hofman 2022) and persists in soils with a half-life of 100–400 days (Karlsson et al. 2016; EU 2018). Thus, to capture potential long-term effects, we extended the duration of the test procedure from 28–56 days, potentially leading to the emergence of effects overlooked in the standard test duration. In addition, we incorporated traditional soil microbiology techniques, including measurements of soil respiration, microbial biomass and enzyme activity to gain insight into potential fungicide effects on microbial activity, biomass and the C-, N- and P-cycles. We hypothesized that (I) the abundance of functional genes would indicate potential boscalid effects clearer than the standard test (Nitrogen Transformation Test, OECD 216) and that (II) the minimum test duration of 28 days may be insufficient to detect long-term effects of the persistent fungicide.

## Materials and methods

### Soil selection

We used LUFA 2.3 standard soil from LUFA Speyer (Germany). The soil properties (Table SI1) meet all requirements of the OCED 216 guideline (sand content 50–75%, pH 5.5–7.5, microbial biomass > 1% of soil organic C). The soil is a sandy loam that has not been fertilized or treated with pesticides in the four years prior to sampling. Fresh topsoil (0–20 cm depth) was sampled on the 2nd of November 2022 and sieved to < 2 mm.

### Experimental design

The experimental design followed the OECD 216 guideline. Boscalid (Merck, Germany) concentrations were based on the Predicted Environmental Concentrations (PEC_soil_,_accu_) for soils under grape cultivars over one year. The used PEC is reported in the list of endpoints for boscalid and was calculated under the assumptions of a soil depth of 5 cm, a bulk density of 1.5 g cm^−3^, a plant interception of 60% and an accumulation factor of 0.95 which leads to a boscalid concentration of approximately 1 mg kg^−1^ dry-weight (dw) soil (EU 2018). Four different fungicide concentrations were applied: 1x PEC (1B), 2x PEC (2B), 5x PEC (5B) and 10x PEC (10B) at 1, 2, 5 and 10 mg kg^−1^ dw soil, respectively. Pesticide-treated soils and fungicide-free controls were replicated four times, resulting in a set of 24 microcosms per sampling day. Each microcosm contained 80 g dw soil and 0.4 g lucerne (*Medicago sativa*) powder with a C/N ratio of 11, to avoid microbial C and N limitations during the incubation. Moreover, we setup a soil control treatment, which consisted of 80 g dw soil without lucerne meal. The data of the soil control are not shown, as the values were below the detection limit of qPCR functional gene measurements. Soil and lucerne powder were mixed and pre-incubated for one week in plastic cups at 20 °C and 40% maximum water holding capacity. This was done to avoid possible interferences in the measurements caused by the initial reactivation of the microbial community. Due to the low water-solubility of boscalid (4.6 mg l^−1^) (Lewis et al. 2006), quartz sand was used as fungicide carrier. Two days before the pre-incubation of the soil ended, 0.8 g of quartz sand was added to the empty microcosms. This sand was treated with 200 µl acetone containing the different boscalid concentrations, while control soils received untreated sand. After pre-incubation and complete acetone evaporation (48 h), the lucerne-soil mixtures were poured from the plastic cubs into the microcosms, which contained the boscalid-coated sand. The soil in each microcosm was thoroughly mixed with the sand to achieve a homogeneous distribution of the fungicide. Microcosms were then incubated in the dark at 20 °C for a maximum of 56 days. Day 56 was chosen as maximum duration, as this is double of the minimum duration (28 days) and still within the 14-days testing scheme after Day 28 of the OECD 216. Destructive sampling of the microcosms was performed on days 0, 3, 7, 14, 21, 28, and 56. Soil samples were stored for further analytics at −20 °C and aliquots for DNA extraction were stored at −80 °C.

### Soil respiration

In the four microcosms with the longest incubation time (56 days), CO_2_ production was monitored throughout the incubation period via CO_2_ traps according to ISO 16072:2011-09. Vials containing 2 ml of 1 M sodium hydroxide were attached to the undersides of the microcosm lids as CO_2_ traps. Respiration rates were measured in a 0.5 ml aliquot of sodium hydroxide to which 0.5 ml of 1 M barium chloride and one to two drops of phenolphthalein were added. The colored solution was titrated with 0.1 M hydrochloric acid to the colorless neutralization endpoint. CO_2_ production was measured on days 1, 3, 5, 7, 10, 14, 17, 21, 24, 28, 31, 38, 45, 52, and 56 of the incubation. Results are given as cumulative CO_2_ production in mg CO_2_ g^−1^ dw soil.

### Boscalid residues

Boscalid residues were extracted by the QuEChERS method for pesticides, according to Silva et al. (2019). Briefly, 1 g homogenized fresh soil for each microcosm was mixed with 5 ml deionized water, 10 ml acetonitrile and shaken for 15 min at 900 rpm. To each sample, 6.5 g of the QuEChERS salts (Agilent, USA), containing 1 g sodium chloride, 4 g magnesium sulphate, 1 g trisodiumcitrate dihydrate, and 0.5 g sodium hydrogen citrate sesquihydrate, were added. Samples were then shaken by hand and the heated tubes were cooled in ice. The cooled samples were shaken for 1 min at 900 rpm and centrifuged at 3000 rpm for 5 min at −5 °C. The acetonitrile layers were transferred into 15 ml tubes and 0.5 ml of the upper organic phase were diluted using 0.5 ml of 0.1% formic acid, mixed, and filtered through a 0.2 µm nylon membrane filter before LC-MS/MS analysis. LC-MS/MS consisted of an Agilent 1200 chromatographic system (Agilent, USA) connected to an ESI/QqQ mass spectrometer (Agilent Triple Quad 6410, Agilent, USA).

### OECD 216: Nitrogen transformation test

Soil nitrate was extracted based on the OECD 216 guideline. Briefly, soil equivalent to 5 g dw was mixed with 25 ml 0.1 M potassium chloride solution in a 50 ml falcon tube. The mixtures were shaken horizontally for 60 min at 150 rpm and centrifugated for 30 min at 4560 rpm. The supernatant was filtered, and the resulting liquid phase was used for the photometric measurement. Nitrate was in a dialyzing step with hydrazine sulfate converted to nitrite, which was then colored with sulfanilamide/a-naphthylethylenediamine and measured at wavelength 540 nm with an Evolution II photometer (Alliance, Austria). Nitrogen transformation rates were expressed as NO_3_-N in mg kg^−1^ dw soil day^−1^.

### Microbial biomass carbon

To determine microbial biomass carbon (C_mic_), two 10 g fresh weight soil aliquots from each sample were weighed. One served as control and the other was fumigated in a chloroform atmosphere for 24 h. The non-fumigated and fumigated samples were mixed with 40 ml of 0.5 M sodium sulfate solution, shaken at 200 rpm for 30 min in a horizontal shaker and centrifuged at 4560 rpm for 30 min. After centrifugation, the clear supernatant was filtered through a 20 µm filter tip, diluted 1:4 with deionized water and stored at −20 °C. Carbon content of samples was then measured on a multi N/C analyzer 2100S (Analytic Jena AG, Germany) and C_mic_ content was calculated by differences in carbon contents between fumigated and non-fumigated samples using the factor of 0.45 according to Joergensen (1996).

### Enzyme activities

Potential activities of the enzymes β-glucosidase (EC 3.2.1.21), β-xylosidase (EC 3.2.1.37), N-acetyl-β-glucosaminidase (EC 3.2.1.52) and acid phosphatase (EC 3.1.3.2) involved in the C, N and P cycles, respectively, were determined with a substrate solution for each enzyme containing the fluorescent 4-methylumbelliferon substrate according to Marx et al. (2001). Briefly, 1 g fresh weight soil was suspended in 50 ml distilled water and sonicated for 120 s at 50 J s^−1^. Fifty µl of the soil solution was transferred into microplate wells in triplicate, followed by 50 µl MES-buffer (pH 6.1) and 100 µl of 1 mM enzyme specific substrate solution. Microplates were pre-incubated for 30 min at 30 °C. Fluorescent measurements were performed after 0, 30, 60, 120 and 180 min at wavelength 360/480 nm (excitation/emission) via a fluorescence microplate reader (FLx800, BioTek Instruments Inc., USA). Between measurements the plates were incubated in the dark at 30 °C. In addition, plates for standard curves, containing concentrations of 0, 0.5, 1, 2.5, 4 and 6 µM 4-methylumbelliferon were measured at each time point.

### DNA extraction and qPCR measurement

DNA was extracted from 0.5 g fresh weight soil following the manufacturer’s protocol of the FastDNA^TM^ SPIN Kit for Soil (MP Biomedicals, USA). As a protocol modification, the SPIN Filter washing step was performed three times. Extracted DNA was photometrically quantified via a NanoDrop^TM^ ND 2000c Spectrophotometer (Thermo Fisher Scientific, Germany), diluted with pure water to 5 ng µl^−1^ templates for qPCR measurements and stored at −20 °C.

The quantification of different target genes was carried out via a 7500 Fast Real-Time PCR System from Applied Biosystems (Germany) using the SYBR Green PCR master mix (Applied Biosystems, Life Technology). Primer sequences and temperature programs are shown in Table SI2. Every SYBR Green reaction contained 7.5 µl SYBR Green, 0.75 µl of each primer (5 µM) and 0.375 µl T4gp32 (MP Biomedicals, USA). For quantification, 1 µl template and 4.625 µl sterile water were used for the bacteria and archaea (16S rRNA) genes, 1.5 µl template and 4.125 µl H_2_O for ITS and 2 µl template and 3.625 µl H_2_O for the different functional genes. The qPCR efficiencies ranged from 90–110%. For quantification, a dilution series of standard DNA ranging from 10^8^ copies µl^−1^ to 10^2^ copies µl^−1^ was used.

### Data analysis

We used the statistical software R 4.3.0 (R Core Team 2023) for statistical data analysis. We performed one-way analysis of variance on the response variables (C_mic_, enzyme activities and gene abundances), with the treatment (boscalid concentration) as fixed effect. For CO_2_ production, we applied a linear mixed effect model using the “nlme” package (Pinheiro J, Bates D, R Core Team 2023) with the microcosm ID as random effect. For each timepoint, we performed a one-way Dunnett’s Test to compare the differences of each treatment to the control. We crossed the boscalid treatments (concentration level; *TrT*) with a dummy factor *Con* (*ConVSTrT*; (Piepho et al. 2006).

## Results

### Boscalid residues

**Fig. 1:**
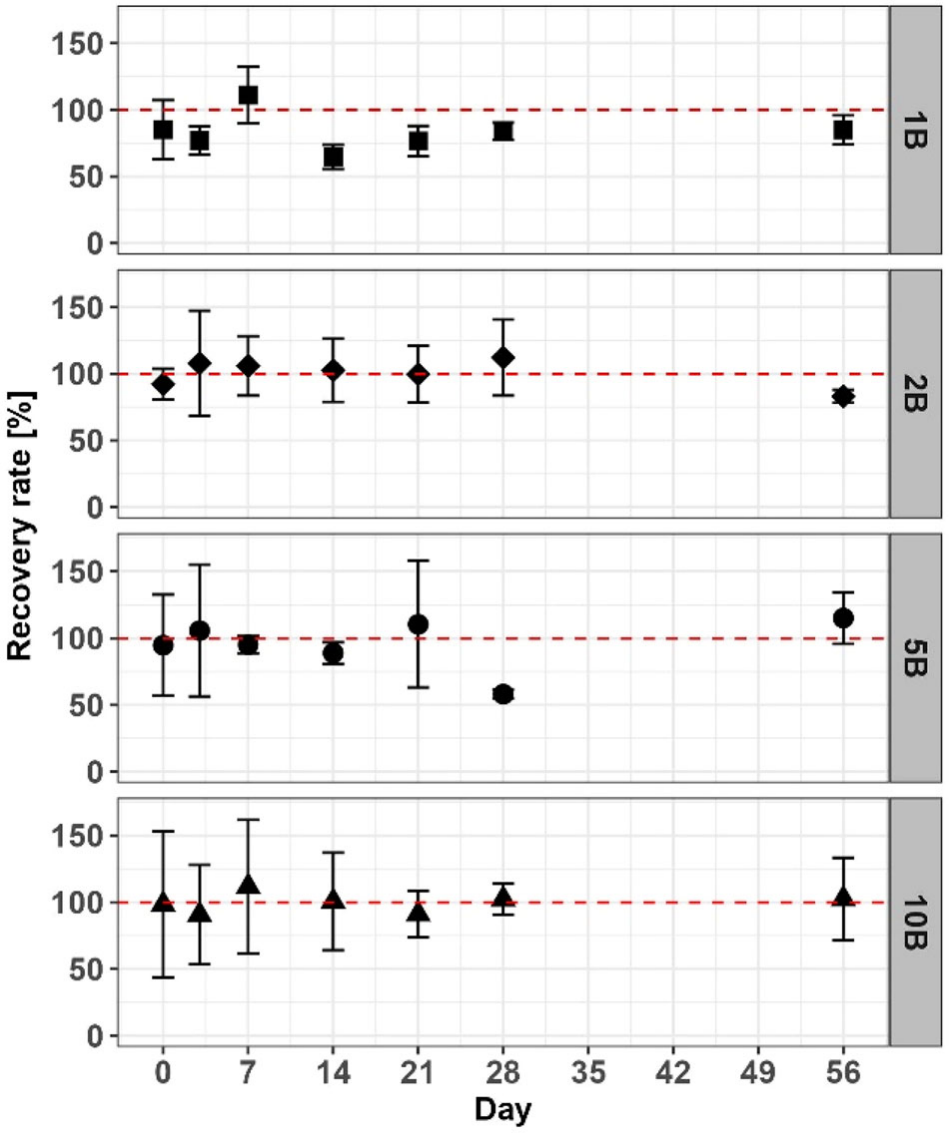
Recovery rate of boscalid (B) in the different treatments (1xPEC: 1B, 2xPEC: 2B, 5xPEC: 5B and 10xPEC: 10B) as percentages of the nominal spiked concentrations (red dotted line). Depicted are the mean and standard deviation of four replicates

During the experiment, boscalid recovery rates of nominal spiked concentrations varied around a mean of 100%, with deviations of 50–150% in the first 7 days (Fig. 1). Even at the end of the incubation no fungicide dissipation was detectable (Table SI3). Due to repeated measurements below the limit of quantification, data point 5B on day 28 represents two repetitions (*n* = 2). No boscalid was detected in the untreated soil.

### OECD 216: Nitrogen transformation test

At all sampling times, N transformation in boscalid treated soils remained within a deviation of < 25% compared to control soils (Table 1). The greatest deviation was observed on Day 28 in the 10B treatment, with a reduced nitrate concentration of 15.9%, followed by a stimulation in the 1B treatment. After 56 days, treatment deviations from the control were below 10%.

**Table 1:** Effect of boscalid on soil nitrogen transformation rate (nitrate production) on days 7, 14, 28 and 56 of the incubation, reported as mean values

Soil [days]	Control	1B		2B		5B		10B	
	NO_3_-N [mg kg^−1^d^−1^]	NO_3_-N [mg kg^−1^d^−1^]	% deviation from control	NO_3_-N [mg kg^−1^d^−1^]	% deviation from control	NO_3_-N [mg kg^−1^d^−1^]	% deviation from control	NO_3_-N [mg kg^−1^d^−1^]	% deviation from control
7	2.86	2.90	1.37	2.46	−14.11	2.98	4.12	2.67	−6.80
14	1.92	1.95	1.53	1.85	−3.62	2.21	15.06	2.16	12.59
28	1.63	1.87	14.64	1.52	−6.88	1.64	0.85	1.37	−15.88
56	1.17	1.26	7.86	1.21	3.19	1.23	4.94	1.08	−7.34

Percentage deviation from the control indicates stimulation (+) or inhibition (-) of the boscalid-treated soils

### Cumulative CO_2_ production

**Fig. 2:**
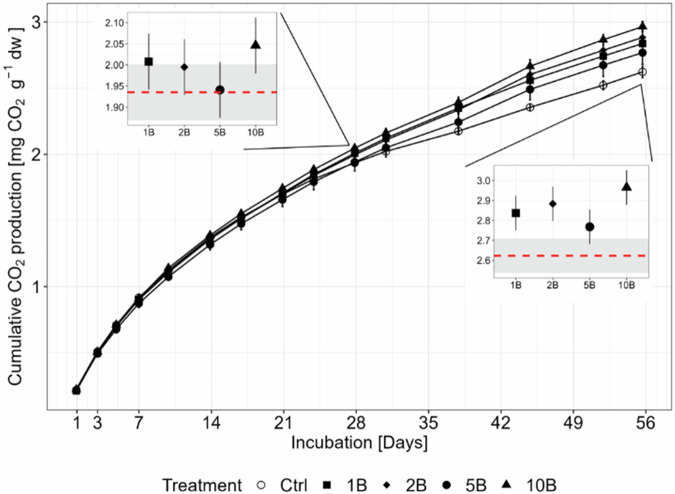
Cumulative CO_2_ production over 56 days in response to different boscalid (B) concentrations (1xPEC: 1B, 2xPEC: 2B, 5xPEC: 5B and 10xPEC: 10B). The embedded insets focus on days 28 and 56 and indicate the differences between treatments and control. Symbols represent estimated marginal treatment means with 95% confidence interval as error bars. The red dotted line represents the estimated marginal control mean with 95% confidence interval as grey rectangles

During the first phase of the incubation (until Day 28), cumulative CO_2_ production in boscalid-treated soils and the control were similar (Fig. 2). At Day 28, the 10B treatment had a significantly higher cumulative CO_2_ production of 5.7% in comparison to the control soil (*p* < 0.01). After 56 days, all boscalid-treated soils were significantly and on average 9.2% higher in CO_2_ production than the control (1B, 2B and 10B *p* < 0.001; 5B *p* < 0.01).

### Microbial biomass carbon

The C_mic_ content decreased over the experimental duration from around 350–540 µg g^−1^ at Day 0 to around 270–370 µg g^−1^ at Day 56 (Fig. SI1). Significantly higher C_mic_ content in comparison to the control was found in treatment 10B at Day 21 (+21.1%, *p* < 0.01) and 5B and 10B at Day 28 (+23.9%, *p* = 0.015; +27.9%, *p* < 0.01).

### Activities of enzymes involved in C, N and P cycles

**Fig. 3:**
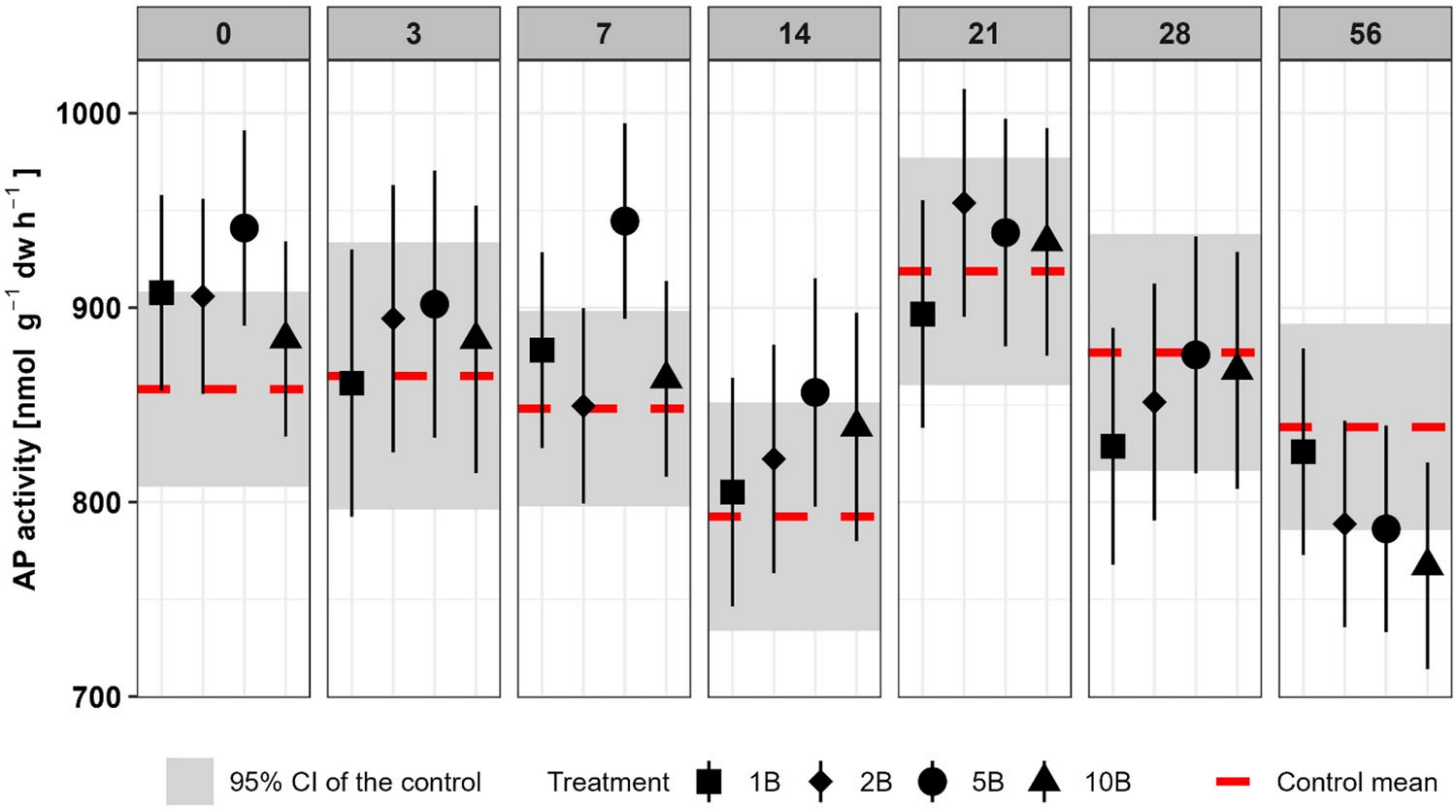
Acid phosphatase (AP) activities on all sampling days. Symbols indicate estimated marginal means with 95% confidence interval as error bars of boscalid (B) treated soils (1xPEC: 1B, 2xPEC: 2B, 5xPEC: 5B and 10xPEC: 10B). The red dotted lines indicate the estimated marginal mean and the grey rectangles the 95% confidence intervals of the control soil

Different enzymes exhibited deviations from the control toward higher activities in boscalid-treated soils within the first week of incubation (D3: BG 5B + 11.2% *p* < 0.05; D7: XYL 5B + 14.6% *p* = 0.087, NAG 5B + 17.6% *p* = 0.078, AP 5B + 11.4% *p* < 0.01; Fig. 3, SI2). In addition, at Day 56 single treatments showed a reduced BG and XYL activity (BG: 1B −9.31% *p* = 0.056; 2B −14.5% *p* < 0.01; XYL: 2B −18.7% *p* < 0.01; Fig. SI2). However, these trends were generally not related to the boscalid concentration. The only exception was the activity of acid phosphatase (AP) at Day 56, which decreased slightly with increasing boscalid concentration, from treatment 2B (−5.9%) to 5B (−6.3%), and was significantly lower than the control in the 10B treatment (−8.5%; *p* < 0.05; Fig. 3).

### Abundances of bacterial and archaeal 16S rRNA and ITS region copy numbers

**Fig. 4:**
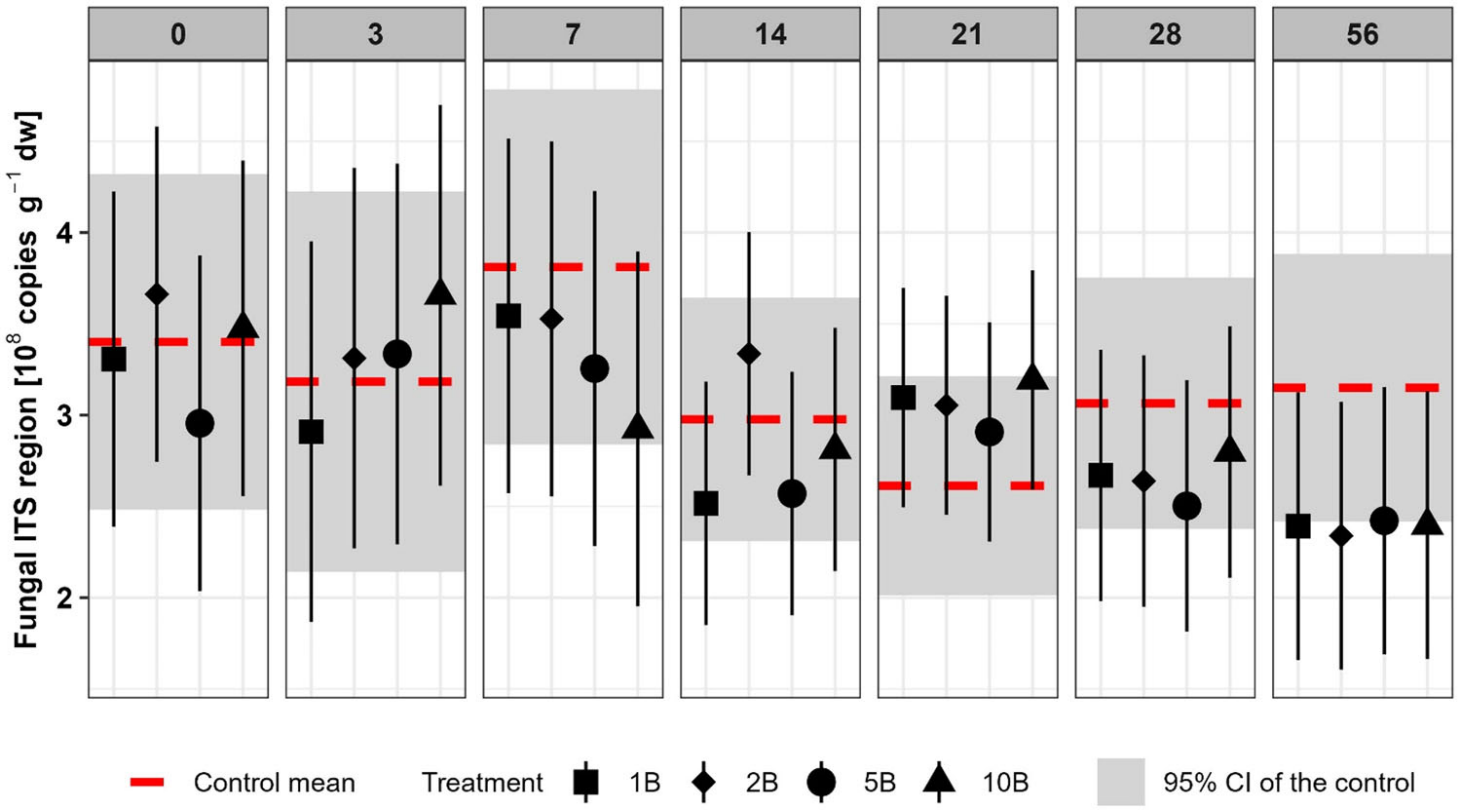
Fungal abundance based on rDNA ITS region copy numbers on all sampling days. Symbols indicate estimated marginal means with 95% confidence interval as error bars of boscalid (B) treated soils (1xPEC: 1B, 2xPEC: 2B, 5xPEC: 5B and 10xPEC: 10B). The red dotted line indicates the estimated marginal mean and the grey rectangle the 95% confidence interval of the control soil

In the early phase of the incubation (up to Day 28) boscalid did not affect the abundance of bacteria and archaea based on 16S rRNA genes, and fungi based on the ITS region (Fig. SI3, 4). However, all treatments declined in fungal ITS region copies compared to the control soil on Day 56 (1B: −24.1%; 2B: −25.8% *p* = 0.1; 5B: −23.1%; 10B −23.9%; Fig. 4). This trend was not related to the concentration of boscalid. The overall abundance of bacterial 16S rRNA gene copies declined from around 2.30 × 10^10^ copies g^-1^ (±7.24 × 10^9^) at Day 0 to 1.22 × 10^10^ copies g^-1^ (±2.16 × 10^9^) at Day 56 (Fig. SI3a). No effects were found for the 16S rRNA archaeal gene abundance (Fig. SI3b).

### Abundance of functional genes

Analyses of the functional genes encoding enzymes involved in the N and P cycles found specific, but not dose-dependent, responses to the fungicide boscalid. The abundances of the nitrogen fixing *nifH* gene and the archaeal ammonia oxidation gene (*amoA* AOA) showed no deviations of > 25% from the control (Fig. SI4, SI5a). In contrast, the bacterial ammonia oxidation gene *amoA* AOB exhibited deviations exceeding 25%, although not significantly (Fig. SI5b). Deviation of *amoA* AOB was −32.8% in the 10B treatment on Day 28 and further declines of −29.5 and −42.7% in the treatments 5B and 10B, respectively, were detected on Day 56 (Fig. SI5b). Similarly, the denitrification genes *nirK* and *nirS* exceeded the 25% threshold, but results did not significantly differ from the control (Fig. SI6). On Day 28, the deviations for *nirK* were −33.8 and −37% in treatments 1B and 5B, respectively, and on Day 56, −40.1% in treatment 5B (Fig. SI6a). For *nirS*, deviations were −42.3 and −34.2% in treatments 1B and 5B, respectively, on Day 28 and on Day 56 −28.3%, −20.1%, −23.8% and −24.1% in treatments 1B, 2B, 5B and 10B respectively (Fig. SI6b).

**Fig. 5:**
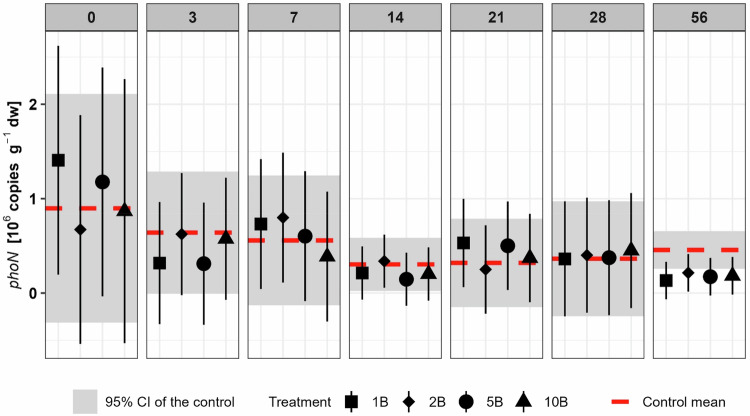
Abundance of *phoN* gene on all sampling days. Symbols indicate estimated marginal means with 95% confidence interval as error bars of boscalid (B) treated soils (1xPEC: 1B, 2xPEC: 2B, 5xPEC: 5B and 10xPEC: 10B). Red dotted lines indicate the estimated marginal mean and the grey rectangles the 95% confidence interval of the control soil

Additionally, copy numbers of the acid phosphatase gene (*phoN*) (Fig. 5) were generally reduced in the presence of boscalid after an incubation period of 56 days, which was unrelated to the boscalid concentration (1B: *p* = 0.01; 2B: *p* = 0.07; 5B: *p* = 0.03; 10B: *p* = 0.04). The reductions relative to the control were substantial, with deviations of −70.7%, −52.9%, −61.9% and −59.8% in the treatments 1B, 2B, 5B, and 10B, respectively.

## Discussion

### OECD 216: Nitrogen transformation test

Our experiment, followed the OECD 216 N transformation test guideline (OECD 2000), which is the currently required test for soil microorganisms in the ERA of pesticides. This test evaluates whether a pesticide causes unacceptable long-term effects (defined as > 25% deviation from the control) on nitrogen transformation by soil microbiota. The deviation criterion (>25%) for the N transformation was not exceeded on any sampling date in the boscalid-treated soils, particularly on Day 28, when it is usually decided whether to continue the test or not. In this regard, the results of our study align with the EU (2018) report on boscalid, which found no deviations from the control greater that 25% at Day 28, up to a concentration of 8 mg kg^−1^, which is close to our 10B treatment. In addition, boscalid showed no dissipation over the 56-day experimental period, which is in line with reported DT_50_ values in sandy soils of approximately 100–400 days (Karlsson et al. 2016; EU 2018). Consequently, the results of our study conclude that boscalid does not have any long-term effects on soil microorganisms according to the current regulatory approach.

### Effect of boscalid on respiration and biomass

Fungicides are known to affect microbial respiration and biomass. Ullah and Dijkstra (2019) showed in their meta-analysis that the type of fungicide determines whether it has an inhibitory or stimulatory effect. In the meta-analysis boscalid showed to have an inhibitory effect on soil respiration. Xiong et al. (2014) also found an inhibition on the respiration, which occurred after seven days of the incubation. In contrast to these studies, we found that microbial respiration in boscalid-treated soils was stimulated towards the end of the experiment, from Day 28 up to Day 56. However, unlike Xiong et al. (2014), we followed the OECD guideline by incorporating lucerne powder into the soil, followed by a one-week pre-incubation. The easily accessible nutrient source may have increased the microorganisms’ ability to compensate for a potential pesticide effect in the initial stages of the experiment (Muñoz-Leoz et al. 2012; Aristi et al. 2016). The plant material may have contributed to the stability of the microbial community after pesticide exposure. For instance, increased bacterial diversity is linked to greater stability of soil microbial communities to anthropogenic stress (Osburn et al. 2023). Given the high recovery of boscalid residues, it is unlikely that the observed elevated CO₂ production resulted from pesticide mineralization. This differs from other pesticides, such as glyphosate and MCPA, in which pesticide mineralization can contribute to increased CO_2_ release from the soil (Wang et al. 2016a; Wirsching et al. 2023).

Instead, the elevated CO_2_ production towards the end of the experiment may be linked to the response of the microbial community to the fungicide. Throughout the experiment, we observed a decline in microbial biomass C and bacterial 16S rRNA gene abundance, particularly during the initial stages of the incubation. The decline may have been linked to the dynamics of plant litter degradation in soils. Initially, the easily accessable plant fraction is degraded, while more complex and recalcitrant compounds remain (Poll et al. 2008). As the potential compensatory effect of the lucerne powder was likely exhausted towards the end of the incubation, fungicide effects became apparent (Aristi et al. 2016). Therefore, we argue that the observed increase in CO_2_ production is primarily attributed to two mechanisms induced by the fungicide. The first one could be a stress response of the fungal community. Boscalid, as a succinate dehydrogenase inhibitor (SDHi), blocks cellular respiration performed by mitochondria. Moreover, an inhibition of the succinate dehydrogenase could result in an accumulation of succinate in the mitochondria of fungi. This could have led to an increased formation of reactive oxygen species (ROS) (Duarte Hospital et al. 2023). Jofré-Fernández et al. (2023) has shown that the production of ROS could result in a significant increase in CO_2_ emissions from the soil. The second potential mechanism for the increase in CO_2_ could involve bacteria mineralizing fungal necro-mass. Boscalid could have ultimately induced fungal cell death, as indicated by the decreasing trend in fungal gene abundance. A similar result was previously observed for thifluzamide, another fungicide of the SDHi group (Yao et al. 2022). Bacteria might thus have contributed to the increased CO₂ production by mineralizing necro-mass in the fungicide-treated soils (Álvarez-Martín et al. 2016). However, the absence of an observed increase in 16S rRNA gene abundances suggests that the microbes used the carbon for maintenance and survival mechanisms rather than for growth (Schimel et al. 2007). We speculate that one survival mechanism could involve the production of extracellular polymeric substances (EPS). These highly hydrated polymers have shown to protect microbes from antimicrobial compound such as antibiotics or disinfection agents (Davenport et al. 2014; Costa et al. 2018). Therefore, the production of EPS could have protected the microbes to get in contact with the barley water-soluble boscalid (Lewis et al. 2006). Nevertheless, most of the observed effects were detected on Day 56, which would be overlooked by the current 28-day testing scheme. Consequently, these results support our second hypothesis, which stated that the test duration may be insufficient for the ecotoxicity testing of persistent pesticides such as boscalid.

### Effect of boscalid on microbial enzyme activities

Modifications in potential enzyme activities and functional gene abundances offer insights into alterations in the microbial communities’ ability to perform ecosystem functions, such as nutrient cycling (Nannipieri et al. 2012). Pesticide toxicity could lead to such alterations, for instance, by increasing microbial mortality (Baćmaga et al. 2018). Our enzymatic data indicated mostly a low response to the fungicide addition, especially in the early stages, as only individual and concentration-independent treatments showed temporary stimulatory effects. In contrast, previous studies have found that fungicides such as azoxystrobin (Baćmaga et al. 2015), tebuconazole, and carbendazim (Wang et al. 2016b), and the formulated product Falcon 460 EC (a mixture of spiroxamine, tebuconazole and triadimenol) (Baćmaga et al. 2016) had mostly inhibitory effects on soil enzymatic activities. The lack of response by most of the enzyme activities in our study may have been due to the fact that extracellular enzymes can be stabilized onto organo-mineral complexes in soils where they might be protected from direct contact with the fungicide (Nannipieri et al. 2018; Olagoke et al. 2020). We speculate that stabilized enzymes in the soil, which were already present before the application of the fungicide, made a greater contribution to the overall activity of these enzymes than those that may have been produced only after the application of the fungicide (Knight and Dick 2004). Nevertheless, at the end of the experiment, the activity of acid phosphatases was reduced in the presence of boscalid, and followed a concentration-dependent trend. The lower phosphatase activity could be caused by either the lower enzyme production of fungi, which decreased in gene abundance during the incubation period (Della Mónica et al. 2018), or by a shift towards a bacterial community producing lower amounts of this enzyme without any changes in biomass (Baćmaga et al. 2018; Kalwasińska et al. 2022).

### Boscalid effects on soil functional gene abundances

Measuring the *phoN* gene abundance provided additional insight into our observed decline in AP activity under boscalid treatment. This gene encodes a class A acid phosphatase, which is mostly found in Alpha- and Gammaproteobacteria (Rossolini et al. 1998; Bergkemper et al. 2016). The observed decline in abundance of this gene for all boscalid-treated soils at the end of the experiment can be explained by direct toxic effects of the fungicide on the abundance of *phoN* carrying Alpha- and Gammaproteobacteria (Baćmaga et al. 2021; Meyer et al. 2024). However, we speculate that boscalid could also indirectly reduce the abundance of these taxa. Some organisms of both phyla are capable of sustaining themselves in a facultative endofungal association (Steffan et al. 2020). The majority of reported endobacteria have lost their ability for independent survival, thereby completing their life cycles using fungal cytoplasm as habitat (Deveau et al. 2018; Zhou et al. 2022). Since the fungal ITS region gene abundance trended down due to boscalid, fungal cell death might have resulted in endobacterial death, which would explain the observed reduction in *phoN* gene abundance.

With respect to nitrogen cycling, our findings indicate that none of the N-cycling genes were significantly affected by the boscalid treatment. However, we observed deviations in N-cycling gene abundances at Day 28 and 56, which exceeded the > 25% deviation criterion from the OECD guideline. Previous studies have found that pesticide exposure can affect genes involved in N-cycling, for example; the *nifH* gene for N-fixation (Walder et al. 2022), the ammonium monooxygenase (*amoA* AOA/AOB) genes for nitrification (Feld et al. 2015; Karas et al. 2018) and *nirK* or *nirS* genes for denitrification (Fang et al. 2020). In case of boscalid, Han et al. (2022) found a reduction in both *nifH* and *amoA* gene abundances. In contrast to the aforementioned studies, we induced a nutrient-rich environment by the incorporation of lucerne material in the soil. Organic soil amendments with a narrow C/N ratio favour N-cycling microorganisms by providing sufficient amounts of nitrogen and carbon (Masunga et al. 2016). We speculate that potential negative effects of boscalid on N-cycling organisms were ‘masked’ by the favorable environmental conditions. This conclusion is supported by the nitrate data obtained from the OECD test. However, we cannot rule out the possibility that N-cycling organisms could be negatively affected after a longer incubation period. It is possible that direct interactions between N and P cycles, for instance due to a reduction in P availability, may indirectly result in a reduction in the abundance of N-cycling organisms and may lead to the disruption of soil functionality (Sun et al. 2015).

The objective of incorporating lucerne powder into the soil is to mitigate C starvation during the test (OECD 2000). However, the plant material may be the reason for the observed smaller changes in the abundance and function of soil microorganisms during the first 28 days of the incubation. The readily available carbon compounds of the lucerne material may adsorb fungicides and thereby alter their adsorption behavior onto organo-mineral complexes (Loffredo et al. 2024). Similarly, it is possible that these compounds may feed into energy-demanding microbial resistance mechanisms against the fungicide (Aristi et al. 2016). Moreover, the lucerne powder may partly explain why we observed no clear dose-dependent response among the tested microbial indicators, which might differ for other organic amendments with e.g. higher C/N ratios. It is possible that the selected concentrations in the experimental setup are too low to observe a clear dose-dependent response. Studies that have identified a clear dose-dependent relationship, have applied concentrations up to several hundred times the field application rate (Xiong et al. 2014; Baćmaga et al. 2015; Wang et al. 2020). However, the use of such high concentrations might not correspond to the required realistic application scenario of the OECD 216.

### Future perspectives on functional gene use for environmental risk assessment

With the advent of high-throughput quantitative-PCR-based assessments, a significant number of specific primers, including those for functional genes involved in C, N, P, and S cycling have been characterized (Zheng et al. 2018). Although ISO standards for direct DNA extraction and the determination of soil microbial groups via qPCR are available, the ‘freedom of choice’ that comes with the large number of existing primers for different genes presents a challenge to standardization of the method. For instance, we found the *phoN* gene a promising candidate, while previous studies have found genes, for example the *amoA* genes, as potential new biomarkers (Karas et al. 2018). Further testing is required to validate a range of functional genes from different nutrient cycles, with the aim to provide a more accurate representation of soil microorganisms in the ERA of pesticides.

In the OECD 216 guideline, the addition of plant material with a C/N ratio between 12 and 16 is a prerequisite for testing. It is worth noting that in the absence of plant material in our soil control treatment, the majority of functional genes were below the limit of detection. Future testing of functional genes should, in this regard, follow the OECD guideline. However, the incubation period should be extended, as the results indicated that a 28-day incubation period may be insufficient to detect long-term effects of persistent pesticides. To enhance the efficacy of the molecular method, it is recommended that Day 56 be included in the testing schedule. In addition, the OECD 216 guideline defines as threshold a deviation criterion of > 25% of the untreated soil. It is questionable if this is transferable to the molecular method, as we found deviations > 25% in the abundances of *nirK*, *nirS* and *amoA* AOB genes. However, these differences were not statistically significant. We recommend significance as decision criterion for functional genes as suggested before by Pedrinho et al. (2024).

## Conclusion

The OECD 216 test is currently the mandatory test for soil microorganisms in the Environmental Risk Assessment of pesticides. It is therefore necessary to enhance the protection of these organisms. The present study demonstrated that functional gene analysis, in particular the *phoN* gene, is capable of detecting long-term boscalid effects on soil microorganisms that would otherwise be overlooked. Furthermore, our results suggest that the minimum runtime of 28 days may not be sufficient to detect long-term effects of persistent pesticides such as boscalid. Therefore, further research is required to identify sensitive functional genes involved in different nutrient cycles that can be used to improve the protection of soil microorganisms. Moreover, the testing scheme should be modified to include an additional time point in order to more accurately assess the long-term effects of persistent PPP. In this context, further studies could elaborate on testing other types of organic amendments, in addition to lucerne, to possibly include them in the testing guideline.

## Supplementary information

Supplementary information

## Data Availability

Data is provided within the manuscript or supplementary information files.
