# Supplementary material for: Quantification of soil microbial functional genes as potential new method in environmental risk assessment of pesticides
Source: Ecotoxicology. 2025 Jun 27;34(7):1312–24. doi: 10.1007/s10646-025-02920-w (PMC12423211; doi:10.1007/s10646-025-02920-w)
Supplement: Supplementary file 1 — Supplementary information [file 10646_2025_2920_MOESM1_ESM.docx]

**Supplementary Information**

**Table SI1** LUFA 2.3 soil properties

| Sand | Silt | Clay | pH | Org. carbon | Nitrogen |
| --- | --- | --- | --- | --- | --- |
| [%] | [%] | [%] | 0.01 M CaCl_2_ | [% C] | [% N] |
| 59.7 ± 0.8 | 33.3 ± 1.3 | 7.0 ± 1.0 | 6.0 ± 0.4 | 0.66 ± 0.05 | 0.08 ± 0.01 |

**Table SI2** Quantitative PCR primer sequences and run conditions

| **Target sequence** | **Primer** | **Run conditions** | **References** |
| --- | --- | --- | --- |
| 16S rRNA gene | 341F: CCT ACG GGA GGC AGC AG  515R: ATT ACC GCG GCT GCT GGC A | 600s at 95°C, Cycles (35): 15s at 95°C, 30s at 60, 30s at72°C, 30s at 75°C | López-Gutiérrez et al. (2004) |
| 16S Archaea | 109F: ACK GCT CAG TAA CAC GT  912R: CTC CCC CGC CAA TTC CTT TA | 600s at 95°C, Cycles (35): 15s at 95°C, 60s at 52°C, 60s at 72°C, 30s at 75°C | Lueders and Friedrich (2000) |
| Fungal ITS region  (amplified region: 5.8S and ITS2) | 3F: GCA TCG ATG AAG AAC GCA GC  4R: TCC TCC GCT TAT TGA TAT GC | 600s at 95°C, Cycles (35): 15s at 95°C, 30s at 55°C, 30s at 72°C, 30s at 76°C | Manerkar et al. (2008) and White et al. (1990) |
| *nifH* | F: AAA GGY GGW ATC GGY AAR TCC ACC AC  R: TTG TTS GCS GCR TAC ATS GCC ATC AT | 600s at 95°C, Cycles (40): 45s at 95°C, 45s at 55°C, 45s at 72°C | Rösch et al. (2002) |
| *amoa* AOA | 19F: ATG GTC TGG CTW AGA CG  616R: GCC ATC CAB CKR TAN GTC CA | 600s at 95°C, Cycles (40): 45s at 95°C, 45s at 55°C, 45s at 72°C | Leininger et al. (2006) |
| *amoa* AOB | 1F: GGG GTT TCT ACT GGT GGT  2R: CCC CTC KGS AAA GCC TTC TTC | 600s at 95°C, Cycles (40): 45s at 95°C, 45s at 60°C, 45s at 72°C | Rotthauwe et al. (1997) |
| *nirK* | 876F: ATY GGC GGV CAY GGC GA  1040R: GCC TCG ATC AGR TTR TGG TT | 600s at 95°C, Cycles (35): 15s at 95°C, 30s at 63°C, 30s at 72°C, 30s at 80°C | Henry et al. (2004) |
| *nirS* | Cd3aF: GTS AAC GTS AAG GAR ACS GG  3cdR: GAS TTC GGR TGS GTC TTS AYG AA | 600s at 95°C, Touchdown Cycles (6): 15s at 95°C, 30s start at 63°C, -1°C for each cycle, 30s at 72°C, Cycles (35): 15s at 95°C, 30s at 58°C, 30s at 72°C, 30s at 80°C | Throbäck et al. (2004) |
| *phoN* | FW: GGA AGA ACG GCT CCT ACC CIW SNG GNC A  RW: CAC GTC GGA CTG CCA GTG IDM IYY RCA | 600s at 95°C, Touchdown Cycles (5): 15s at 95°C, 30s start at 65°C, -1°C for each cycle, 45s at 72°C, Cycles (40): 15s at 95°C, 30s at 60°C, 45s at 72°C | Bergkemper et al. (2016) |

M = A/C, S = G/C, R = A/G, Y = C/T, K = G/T, W = C/G/T, V = A/C/G, N = A/C/G/T

**Table SI3** Boscalid (B) concatenations on all sampling days. Displayed as mean concentrations and standard deviations for all boscalid-treated soils (1B, 2B, 5B and 10B)

| **Soil**  **[days]** | **1B** | | **2B** | | **5B** | | **10B** | |
| --- | --- | --- | --- | --- | --- | --- | --- | --- |
|  | Mean concentration  [mg kg^-1^] | Standard deviation  [mg kg^-1^] | Mean concentration  [mg kg^-1^] | Standard deviation  [mg kg^-1^] | Mean concentration  [mg kg^-1^] | Standard deviation  [mg kg^-1^] | Mean concentration  [mg kg^-1^] | Standard deviation  [mg kg^-1^] |
| 0 | 0.85 | ±0.22 | 1.84 | ±0.23 | 4.74 | ±1.90 | 9.84 | ±5.47 |
| 3 | 0.77 | ±0.11 | 2.16 | ±0.79 | 5.28 | ±2.46 | 9.08 | ±3.73 |
| 7 | 1.11 | ±0.21 | 2.12 | ±0.45 | 4.76 | ±0.32 | 11.18 | ±5.00 |
| 14 | 0.65 | ±0.09 | 2.05 | ±0.47 | 4.45 | ±0.40 | 10.07 | ±3.65 |
| 21 | 0.77 | ±0.11 | 1.99 | ±0.43 | 5.52 | ±2.37 | 9.11 | ±1.74 |
| 28 | 0.84 | ±0.06 | 2.24 | ±0.57 | 2.91 | ±0.16 | 10.23 | ±1.16 |
| 56 | 0.85 | ±0.11 | 1.66 | ±0.10 | 5.76 | ±0.97 | 10.25 | ±3.09 |


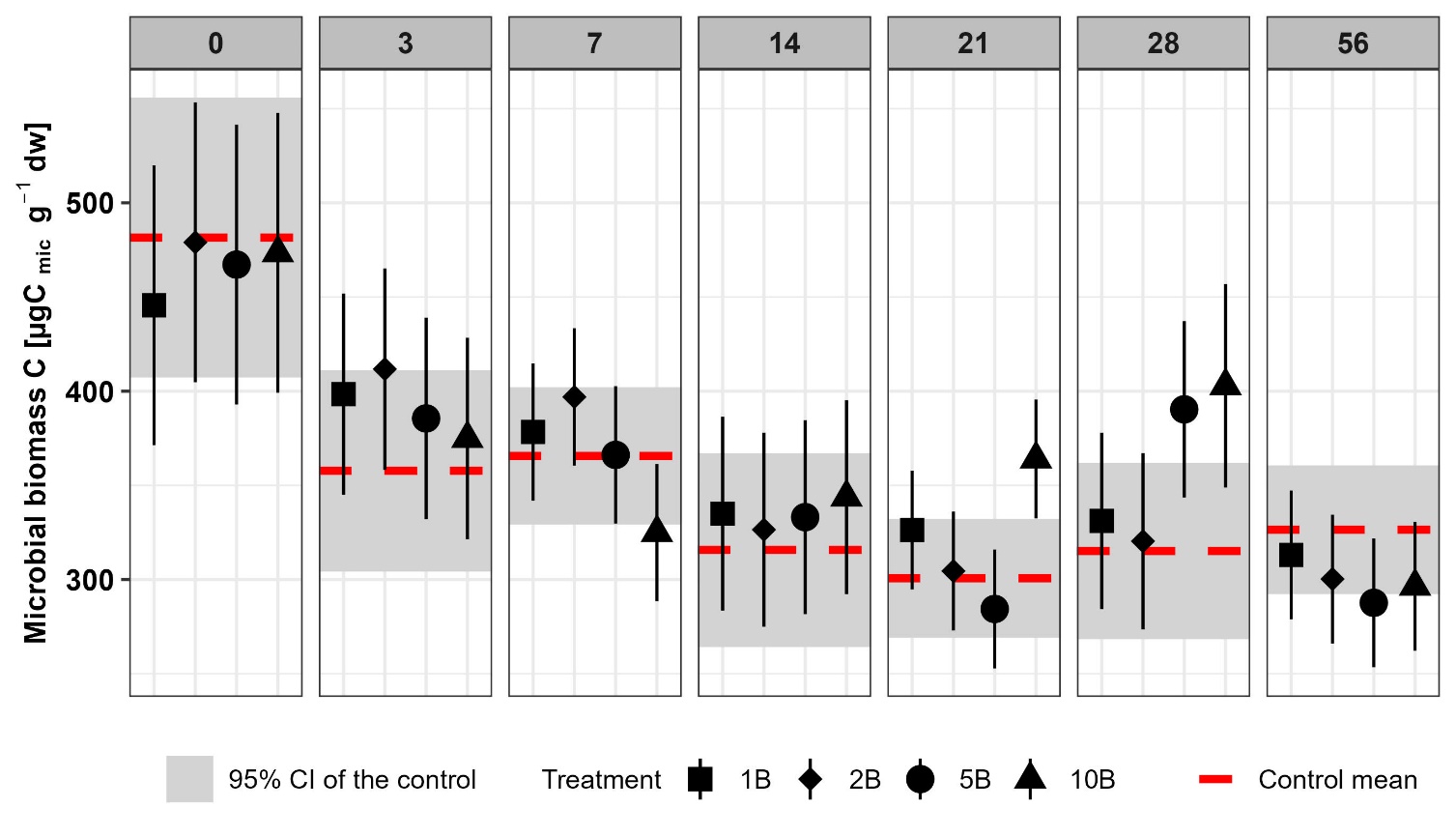


**Fig. SI1** Effect of boscalid on microbial biomass C (C_mic_) on all sampling days. Symbols indicate estimated marginal means with 95% confidence interval as error bars of boscalid (B) treated soils (1xPEC: 1B, 2xPEC: 2B, 5xPEC: 5B and 10xPEC: 10B). Red dotted lines indicate the estimated marginal mean and the grey rectangles the 95% confidence intervals of the control soil

c)

b)

a)


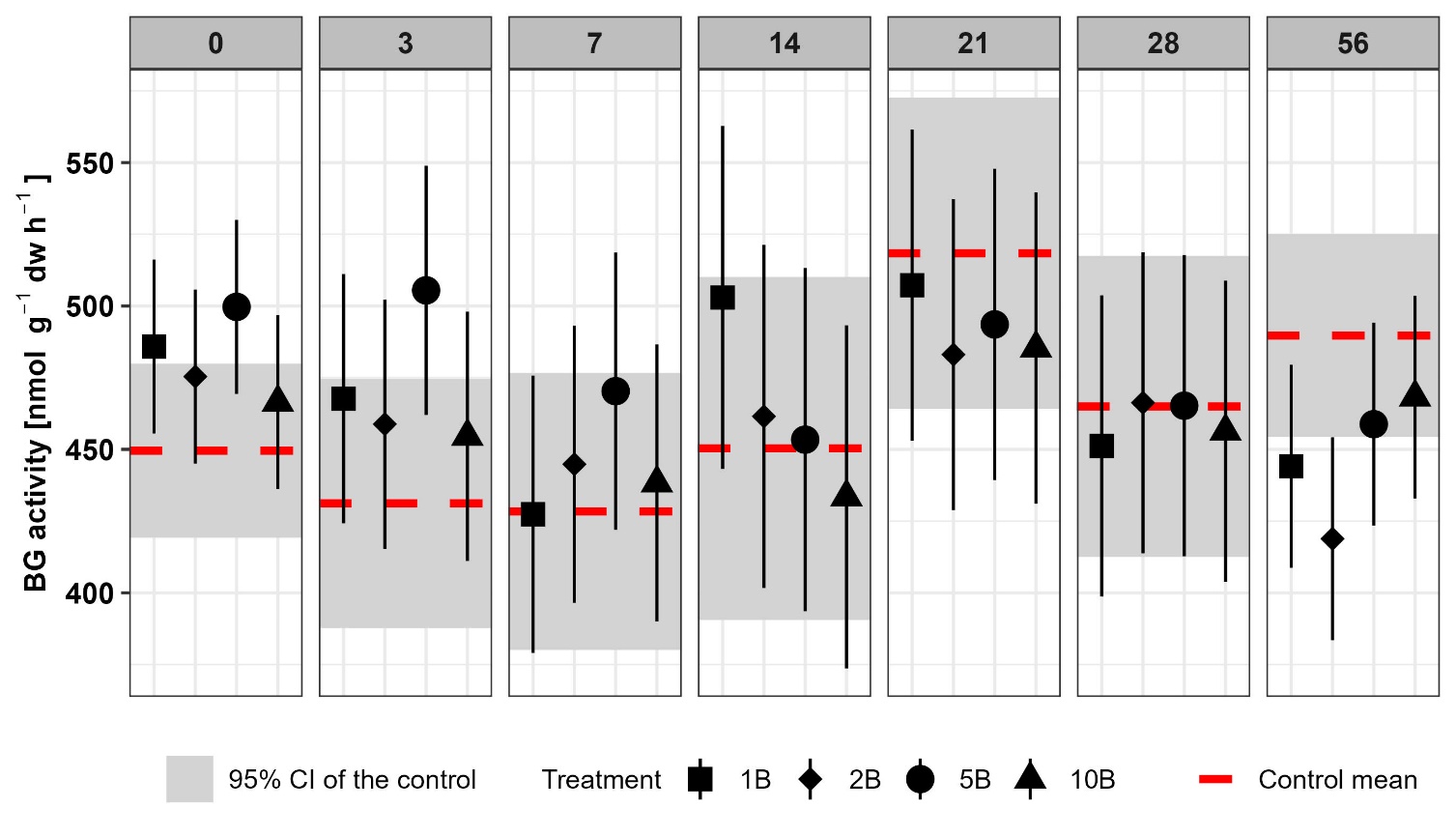

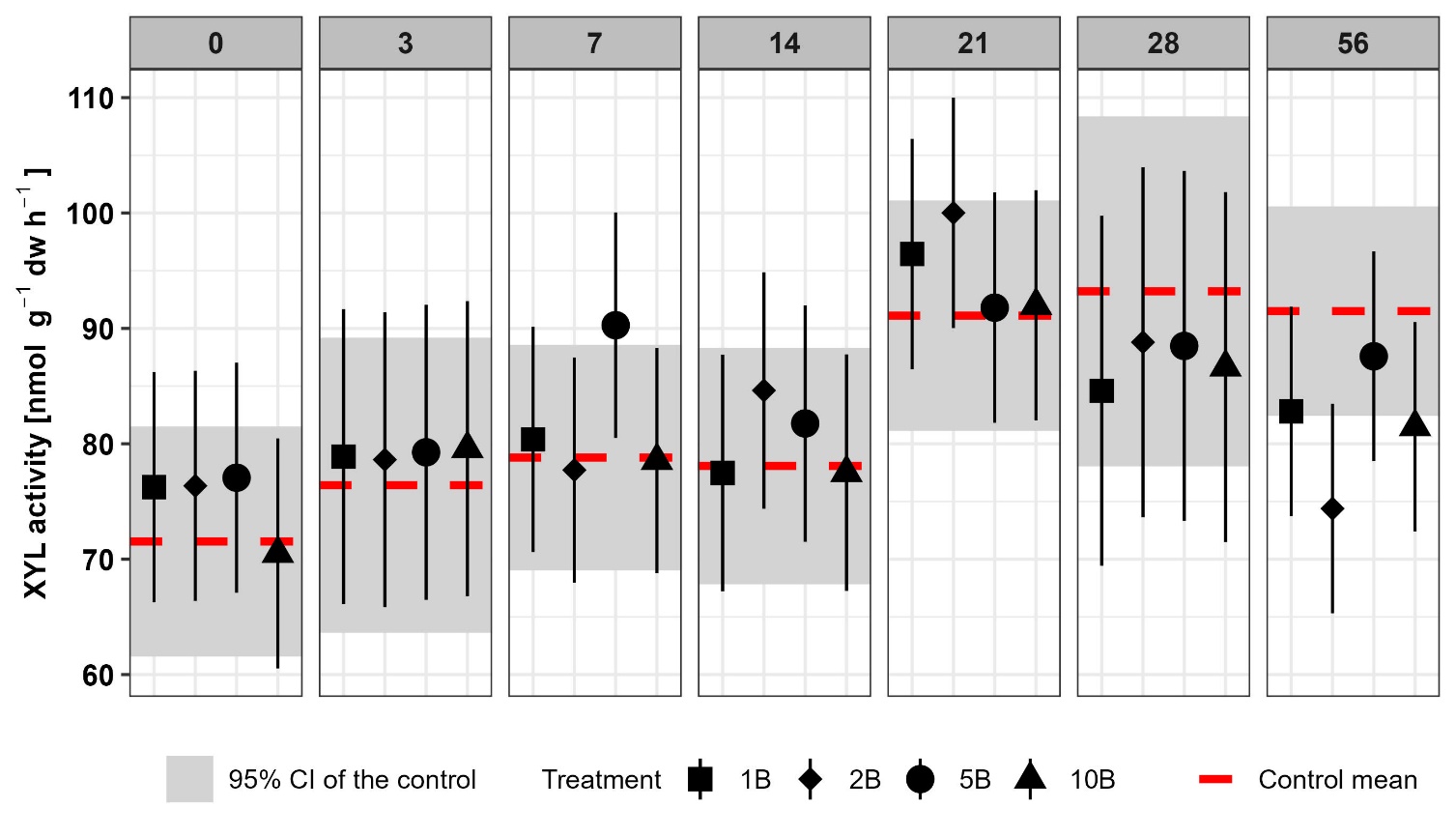

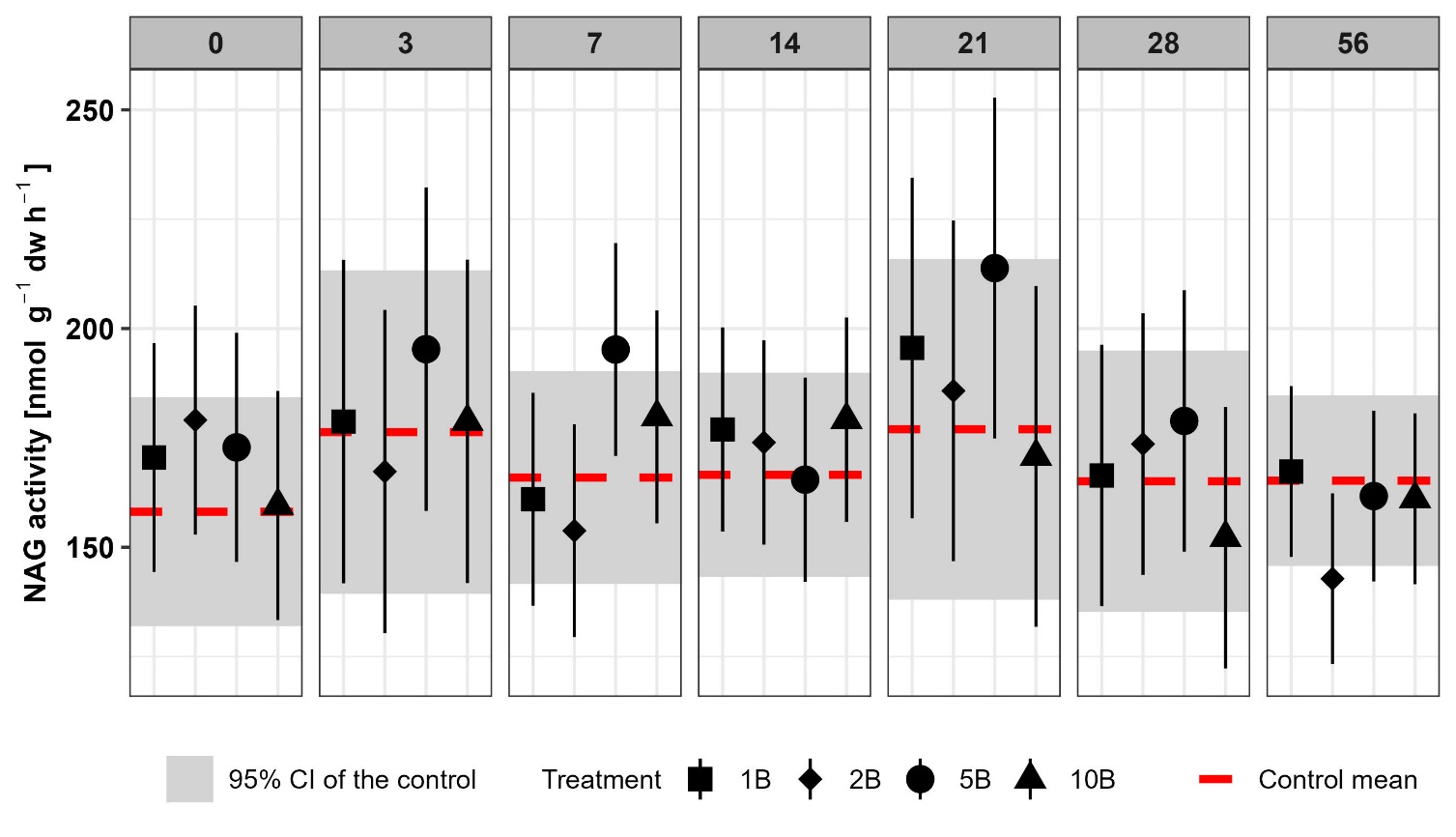


**Fig SI2** Effect of boscalid (B) on enzyme activities of β-glucosidase (a), β-xylosidase (b) and N-acetyl-β-glucosaminidase (c) on all sampling days. Symbols indicate estimated marginal means with 95% confidence interval as error bars of boscalid treated soils (1xPEC: 1B, 2xPEC: 2B, 5xPEC: 5B and 10xPEC: 10B). Red dotted lines indicate the estimated marginal mean and the grey rectangle the 95% confidence interval of the control soil


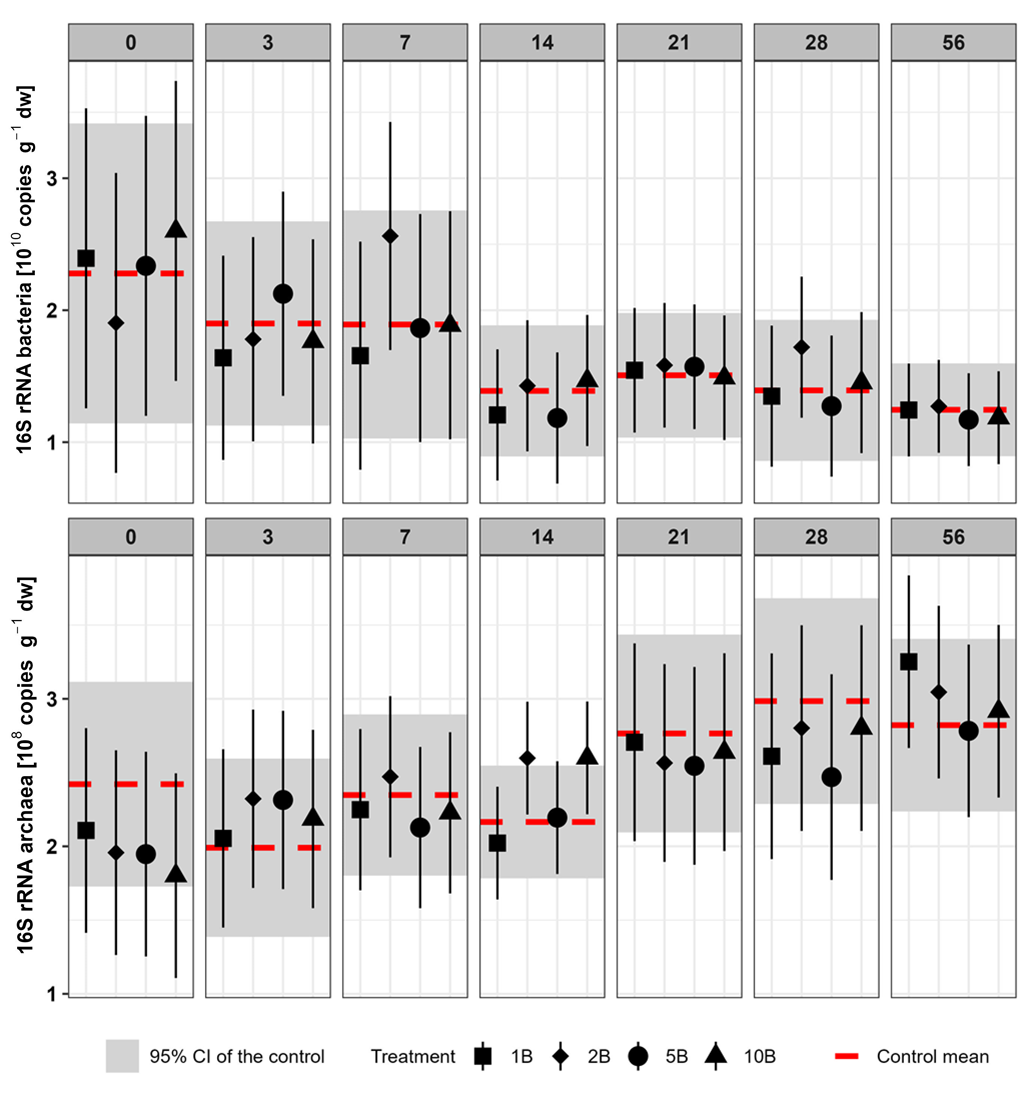


b)

a)

**Fig. SI3** Effect of boscalid (B) on abundances of 16S rRNA bacteria (a) and archaea (b) on all sampling days. Symbols represent estimated marginal means with 95% confidence interval as error bars of boscalid treated soils (1xPEC: 1B, 2xPEC: 2B, 5xPEC: 5B and 10xPEC: 10B). Red dotted lines indicate the estimated marginal mean and the grey rectangle the 95% confidence interval of the control soil


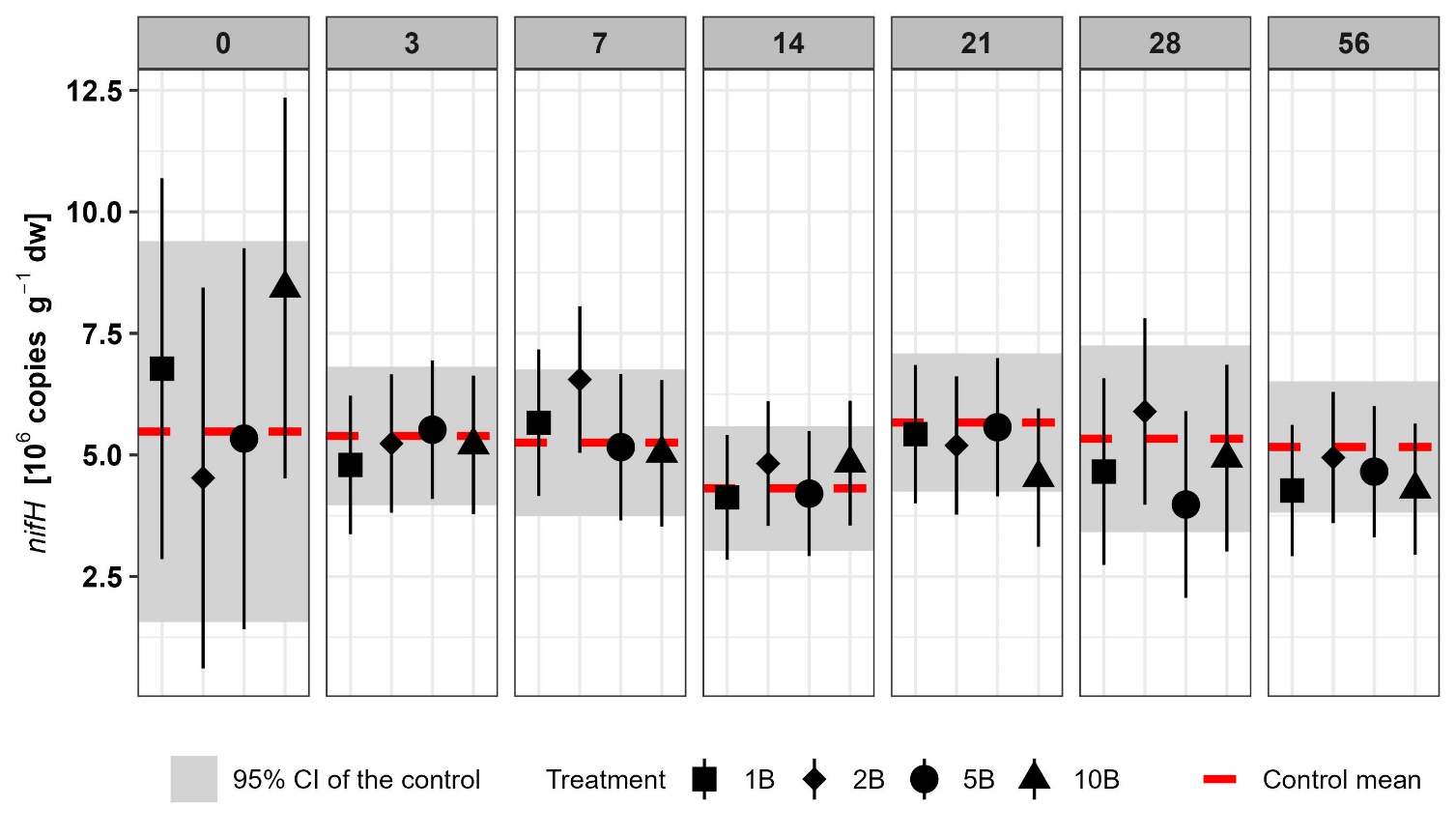


**Fig. SI4** Effect of boscalid (B) on the abundance of *nifH* on all sampling days. Symbols represent estimated marginal means with 95% confidence interval as error bars of boscalid treated soils (1xPEC: 1B, 2xPEC: 2B, 5xPEC: 5B and 10xPEC: 10B). Red dotted lines indicate the estimated marginal mean and the grey rectangle the 95% confidence interval of the control soil
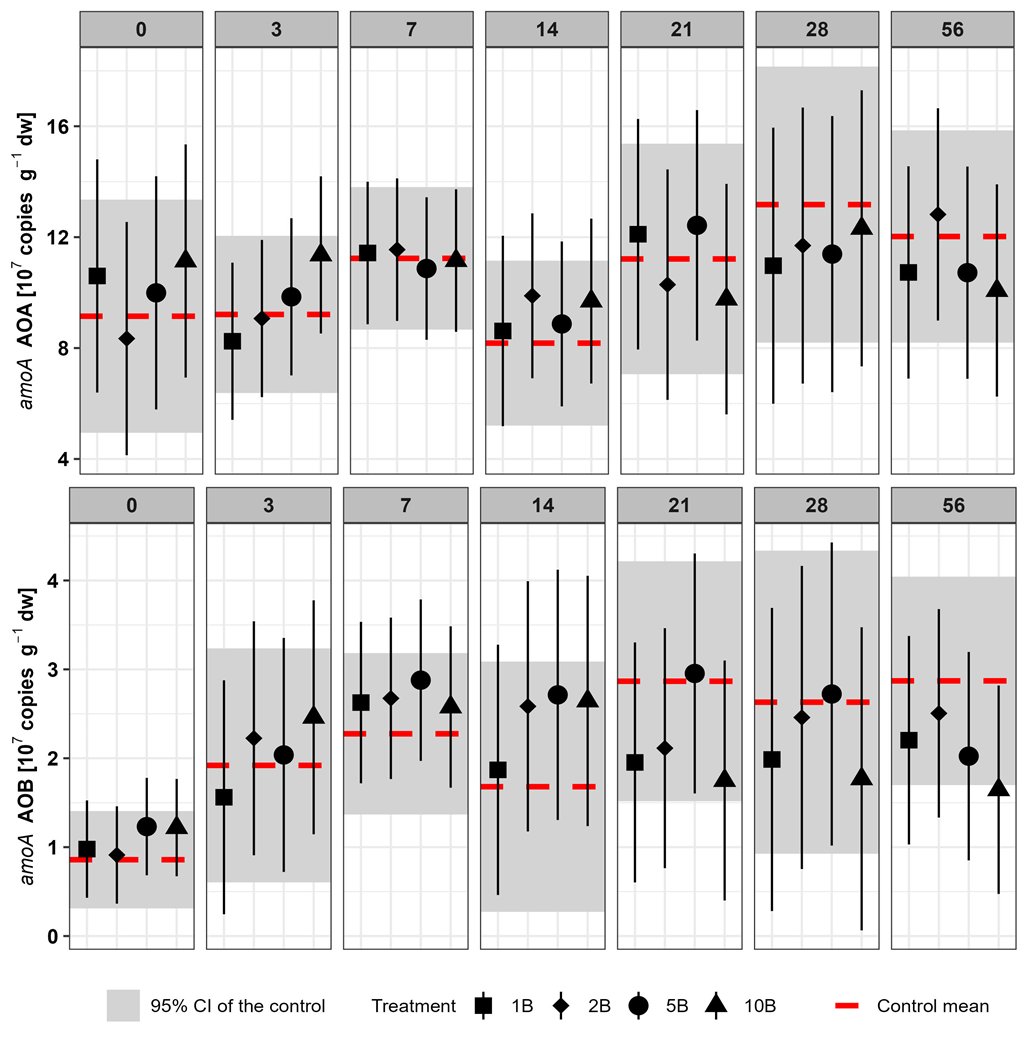


b)

a)

**Fig. SI5** Effect of boscalid (B) on abundances of *amoA* AOA (a) and *amoA* AOB (b) on all sampling days. Symbols represent estimated marginal means with 95% confidence interval as error bars of boscalid treated soils (1xPEC: 1B, 2xPEC: 2B, 5xPEC: 5B and 10xPEC: 10B). Red dotted lines indicate the estimated marginal mean and the grey rectangle the 95% confidence interval of the control soil


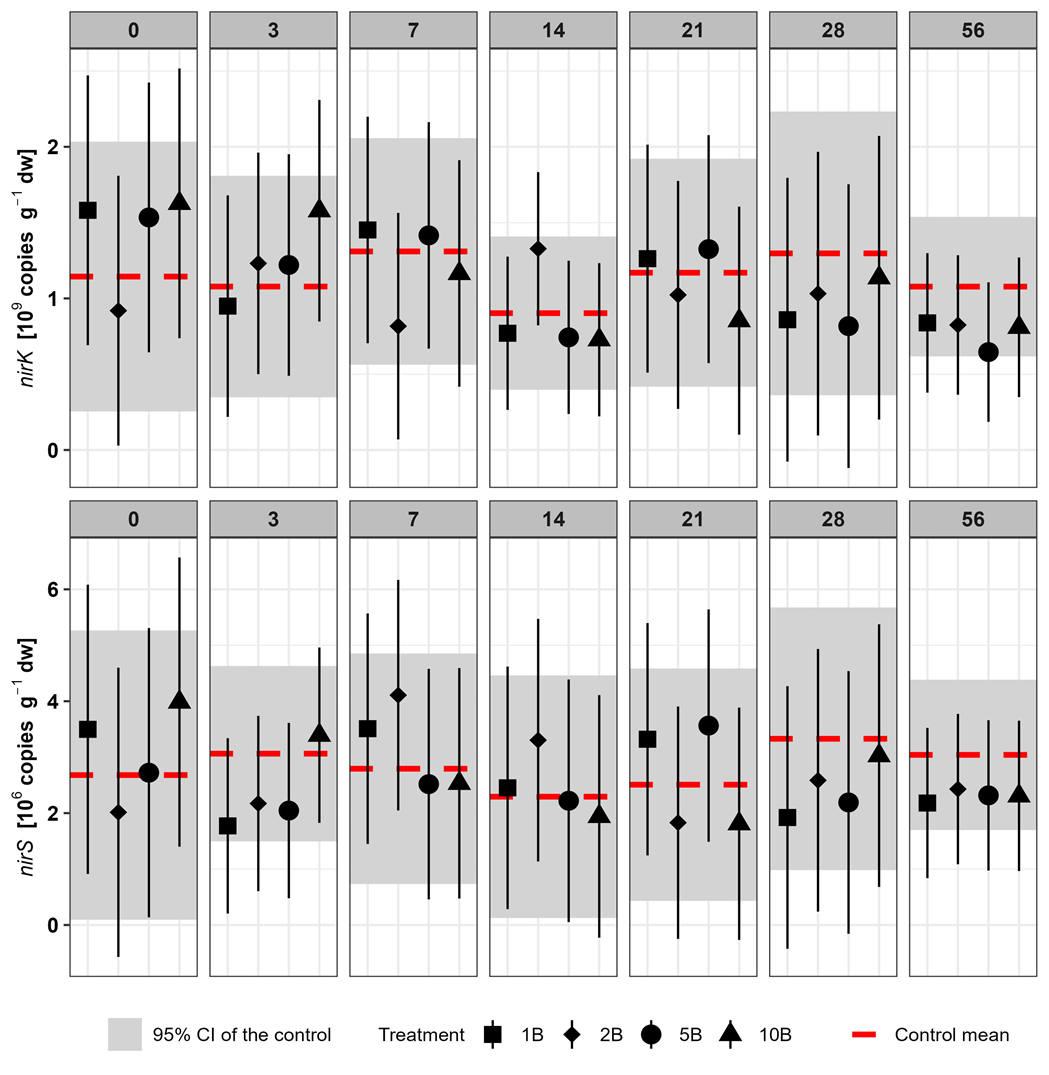


b)

a)

**Fig SI6** Effect of boscalid (B) on abundances of *nirK* (a) and *nirS* (b) on all sampling days. Symbols represent estimated marginal means with 95% confidence interval as error bars of boscalid treated soils (1xPEC: 1B, 2xPEC: 2B, 5xPEC: 5B and 10xPEC: 10B). Red dotted lines indicate the estimated marginal mean and the grey rectangle the 95% confidence interval of the control soil

**References**

Bergkemper F, Kublik S, Lang F, Krüger J, Vestergaard G, Schloter M, Schulz S (2016) Novel oligonucleotide primers reveal a high diversity of microbes which drive phosphorous turnover in soil. J Microbiol Methods 125:91–97. https://doi.org/10.1016/j.mimet.2016.04.011

Henry S, Baudoin E, López-Gutiérrez JC, Martin-Laurent F, Brauman A, Philippot L (2004) Quantification of denitrifying bacteria in soils by nirK gene targeted real-time PCR. J Microbiol. Methods 59:327–335. https://doi.org/10.1016/j.mimet.2004.07.002

Leininger S, Urich T, Schloter M, Schwark L, Qi J, Nicol GW, Prosser JI, Schuster SC, Schleper C (2006) Archaea predominate among ammonia-oxidizing prokaryotes in soils. Nature 442:806–809. https://doi.org/10.1038/nature04983

López-Gutiérrez JC, Henry S, Hallet S, Martin-Laurent F, Catroux G, Philippot L (2004) Quantification of a novel group of nitrate-reducing bacteria in the environment by real-time PCR. J Microbiol Methods 57:399–407. https://doi.org/10.1016/j.mimet.2004.02.009

Lueders T, Friedrich M (2000) Archaeal population dynamics during sequential reduction processes in rice field soil. Appl Environ Microbiol 66:2732–2742. https://doi.org/10.1128/AEM.66.7.2732-2742.2000

Manerkar MA, Seena S, Bärlocher F (2008) Q-RT-PCR for assessing archaea, bacteria, and fungi during leaf decomposition in a stream. Microb Ecol 56:467–473. https://doi.org/10.1007/s00248-008-9365-z

Rotthauwe JH, Witzel KP, Liesack W (1997) The ammonia monooxygenase structural gene amoA as a functional marker: molecular fine-scale analysis of natural ammonia-oxidizing populations. Appl Environ Microbiol 63:4704–4712. https://doi.org/10.1128/aem.63.12.4704-4712.1997

Rösch C, Mergel A, Bothe H (2002) Biodiversity of denitrifying and dinitrogen-fixing bacteria in an acid forest soil. Appl Environ Microbiol 68:3818–3829. https://doi.org/10.1128/AEM.68.8.3818-3829.2002

Throbäck IN, Enwall K, Jarvis A, Hallin S (2004) Reassessing PCR primers targeting nirS, nirK and nosZ genes for community surveys of denitrifying bacteria with DGGE. FEMS Microbiol Ecol 49:401–417. https://doi.org/10.1016/j.femsec.2004.04.011

White TJ, Bruns T, Lee S, Taylor J (1990) Amplification and direct sequencing of fungal ribosomal RNA genes for phylogenetics. PCR Protocols. Elsevier, pp315–322. https://doi.org/10.1016/b978-0-12-372180-8.50042-1
